# Changes in Heat Resistance and Mechanical Properties of Peroxide Cross-Linking HDPE: Effects of Compounding Cross-Linkers

**DOI:** 10.3390/polym17040535

**Published:** 2025-02-19

**Authors:** Shunquan Liu, Run Zhang, Chenchao Fu, Tianshuo Zheng, Ping Xue

**Affiliations:** College of Mechanical and Electrical Engineering, Beijing University of Chemical Technology, Beijing 100029, China; liusq19990325@163.com (S.L.); zhangrun_buct@163.com (R.Z.); fuchenchao1017@163.com (C.F.); 2023210438@buct.edu.cn (T.Z.)

**Keywords:** HDPE, mechanical properties, heat resistance, peroxide cross-linking, two-step preparation process

## Abstract

Due to excellent chemical resistance and impermeability, high-density polyethylene (HDPE) is widely used in petrochemical transportation, product packaging, sports equipment, and marine applications. Yet, with the wide variety of service environments, its mechanical and thermal properties do not meet the demand. In the present study, a compounding cross-linker comprising di-tert-butyl peroxide (DTBP) and triallyl isocyanurate (TAIC) is employed by combining with a two-step preparation process. High-quality cross-linking reactions are achieved for HDPE. In this study, the cross-linking of DTBP is first examined separately. A peak cross-linking degree of 74.7% is achieved, and there is a large improvement in thermal resistance and mechanical properties. Subsequently, the composite cross-linking system of DTBP and TAIC is investigated. The peak cross-linking degree is 82.1% (10% increase compared to DTBP). The peak heat deformation temperature is 80.1 °C (22% increase compared to DTBP). The peak impact strength is 104.73 kJ/m^2^ (207% increase compared to neat HDPE). The flexural strength is 33.6 MPa (22% increase compared to neat HDPE). The results show that this cross-linking system further improves the cross-linking degree, heat resistance, and mechanical properties of HDPE, indicating its potential application in engineering materials for high performance.

## 1. Introduction

HDPE is widely utilized in environmental protection, agriculture, petrochemical transportation, and communication due to its excellent wear resistance, toughness, weatherability, ease of processing, and recyclability [1,2,3,4,5,6,7]. However, compared to other engineering plastics, HDPE has several drawbacks, such as lower heat deformation temperature (HDT) than polyamide (PA) and polyoxymethylene (POM), which limits its use in harsher application situations [8,9,10]. Copolymerization, cross-linking, surface, and blending modifications are frequently used to improve the performance of HDPE [11]. Of these, cross-linking modification not only has a fast processing speed, high production efficiency, and less pollution but it can also effectively improve the mechanical properties of HDPE, heat resistance, and chemical resistance [12,13,14]. Currently, the main cross-linking modification methods for HDPE are peroxide cross-linking (PEX_a_), silane cross-linking (PEX_b_), and radiation cross-linking (PEX_c_) [15,16,17,18]. The cross-linked structure in PEX_b_ is formed by the reaction between the silane cross-linking agent and the HDPE molecular chain, which has a poor cross-linking uniformity. The PEX_c_ method employs electron beams or gamma rays to achieve cross-linking. However, it has high equipment costs and may lead to discoloration and degradation of mechanical properties [19]. PEX_a_ is formed by the reaction of free radicals generated by oxides with HDPE molecular chains to form a cross-linked network [20]. It has the advantages of fast cross-linking speed, uniform cross-linking, and good heat resistance, which makes it the most widely used method in HDPE cross-linking modification.

The cross-linking mechanism of PEX_a_ is based on the thermal decomposition of the cross-linking agent to produce reactive free radicals, which capture hydrogen atoms on the HDPE molecular chain to form a cross-linked structure [21,22,23,24]. The creation of the cross-linked network influences the mobility of the molecular chains and the crystallization process, resulting in changes in a variety of material properties. At the same time, different peroxide cross-linkers and their additives have different effects on the improvement of cross-linking properties.

Ren et al. [25] showed that, using a two-step process with bis (tertbutyldioxyisopropyl) benzenehexane (BIPB) as a peroxide cross-linking agent, the peak impact strength of HDPE after cross-linking at ambient temperature (20 °C) was 78 kJ/m^2^. Meanwhile, the peak impact strength was up to 88 MPa at −10 °C, which was mainly attributed to the increase in the cross-linking degree. The increased cross-linking degree leads to the decrease in the crystallinity (*X_c_*), the size of spherical crystals, and the grain size. The authors suggested that BIPB is more compatible with HDPE and can undergo a more effective cross-linking reaction. However, its thermal stability is relatively poor, resulting in generally lower processing temperatures compared to other peroxide cross-linking agents.

Khonakdar et al. [26] showed that di-tert butyl cumyl peroxide (BCUP) had a significant effect on the melting temperature (*T_m_*), crystallization temperature (*T_c_*), and *X_c_* of HDPE. When the content of BCUP was 3.0%, *T_m_*, *T_c_*, and *X_c_* were reduced to 122.6 °C, 110.2 °C, and 49.5%, respectively. Meanwhile, in terms of thermal stability, the use of a peroxide cross-linking agent such as BUCP had almost no effect on thermal stability. As for the mechanical properties, the tensile strength and yield strength decrease significantly with the increase in BUCP. The authors suggested that BCUP is more compatible with HDPE compared to other cross-linking agents. However, the enhancement of the mechanical properties of HDPE in this study is not significant enough compared to using other cross-linking agents [27]. Moreover, adding BUCP to HDPE did not improve the heat resistance of the HDPE.

Kang et al. [28] showed that the use of DTBP as a cross-linking agent in a one-step process significantly improved properties of HDPE. And the improvement of mechanical and thermal properties of HDPE by DTBP was more significant compared to other cross-linking agents. But the poor compatibility easily leads to insufficient cross-linking reactions. According to the existing research, TAIC has better compatibility with HDPE and can form a better cross-linking network structure. However, its thermal stability and heat resistance are all poor. So, this study explores the system composed of DTBP and TAIC to further improve the quality of cross-linking [29].

In terms of the cross-linking process, peroxide cross-linking of HDPE can usually be achieved by one- or two-step preparation [30]. The one-step method, also known as the Engel method, is characterized by simultaneous extrusion and cross-linking. This means that extrusion and cross-linking are accomplished at elevated temperatures using high-pressure extrusion equipment [31,32,33,34]. Manley et al. [35] studied the effect of different concentrations of peroxide cross-linking agents on the mechanical properties of HDPE using the Engel method. They simultaneously added the cross-linking agent, lubricant, and HDPE for reaction and extrusion. The main drawbacks of this method were that the control of the process parameters was very difficult, the cross-linking reaction was not sufficient, and the material was prone to early degradation.

The two-step approach, also known as the Pont-à-Mousson method, separates the extrusion and cross-linking processes. Specifically, the pre-processed material is first extruded at a low temperature by a screw extruder and then subjected to a cross-linking reaction by a high-temperature heating device. Walter et al. [36] invented a peroxide-cross-linked PE polymer pipe and a method for making it. A pre-cross-linking reaction was performed by adding a modest amount of cross-linking. The main function of the pre-cross-linking reaction is to enhance the melt strength of polymer, ensuring that the pipe molding billet does not collapse before reaching the cross-linking device. Compared to the one-step technique, the two-step method provides more flexible control and a more adequate cross-linking reaction. At the same time, because low-temperature extrusion prevents or only causes micro-cross-linking, the extrusion pressure is substantially lower than in a one-step method [37].

This study focuses on the effect of a compounding cross-linking system consisting of DTBP and TAIC on the mechanical properties and heat resistance of HDPE. Specimens with different ratios of the two cross-linkers were first prepared by a two-step method. Key performance indicators, including HDT, Vicat softening temperature (VST), mechanical properties, cross-linking degree, and *X_c_*, were subsequently evaluated. In addition, this study contributes to an in-depth understanding of the cross-linking mechanism of DTBP and TAIC. It provides a theoretical basis and experimental guidance for optimizing the material properties and process conditions. And it lays a certain foundation for the wide application of cross-linked HDPE in the future.

## 2. Materials and Methods

### 2.1. Materials

High-density polyethylene (HDPE, granular, brand 9680, melt index 6.0 g/10 min, relative molecular mass 27.89 × 10^4^) was purchased from Hanwha Group (Seoul, Korea). The main cross-linker, di-tert-butyl peroxide (DTBP, liquid, purity ≥ 97%), was purchased from Shanghai McLean Biochemical Technology Co., Ltd. (Shanghai, China). Triallyl isocyanurate (TAIC, liquid, purity ≥ 98%) and didodecyl thiodipropionate (DLTDP, powder, purity ≥ 99.7%) were used as an auxiliary cross-linker and a secondary antioxidant, respectively, both of which were purchased from Beijing Chemical Works Co., Ltd. (Beijing, China). Antioxidant 1076 (A1076, C_35_H_62_O_3_, powder, purity ≥ 99.99%, brand 1076) as the main antioxidant was purchased from Shanghai Aladdin Biochemical Technology Co., Ltd. (Shanghai, China). Calcium stearate (CaSt, Ca(C_17_H_35_COO)_2_, powder, calcium content 6.5 ± 0.5%) was used as an internal and external lubricant and purchased from Shanghai Myriad Chemical Technology Ltd. (Shanghai, China).

### 2.2. Preparation of Peroxide-Cross-Linked HDPE Specimens

**Figure 1:**
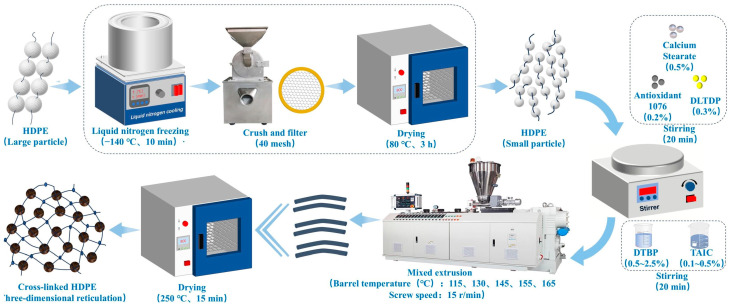
Molding process for peroxide-cross-linked HDPE.

The HDPE particles were first ground into 40-mesh powder using the freeze pulverization process. The powder was stored in a vacuum drying oven (DZF-6050, Beijing Lu Xi Technology Co., Ltd., Beijing, China) at 80 °C for 3 h. The powdered material was then added to a high-speed mixer (MY100, Zhengzhou Zheke Machinery Equipment Co., Ltd., Zhengzhou, China) based on the mass ratio of CaSt:A1076:DLTDP= 0.5:0.2:0.3; swirled and blended for 20 min. Then, DTBP and TAIC were added proportionally to the high-speed mixer to continue stirring and mixing for 20 min. The proportions of the components are shown in Table 1. All component ratios in the table are given in mass fraction (wt%), and the total sum of all component ratios, including HDPE, is 100%. The mixed materials were inserted into a conical twin-screw extruder (EPCT-20, Harper Electric Co., Ltd., Harbin, China) for extruding. The barrel temperature was set as 115, 130, 145, 155, and 165 °C. And the extruder speed was 15 r/min. The extruded specimens were placed in a vacuum oven at 250 °C for 15 min. The detailed preparation process is depicted in Figure 1. The samples were then cut into dumbbell-shaped and rectangular specimens using a cutting tool after heat treatment.

### 2.3. Characterization and Analysis Methods

#### 2.3.1. Measurement of Cross-Linking Degree (Gel Content)

The cross-linking degree was evaluated using a Soxhlet extraction apparatus (Sichuan Shu Glass Co., Ltd., Chengdu, China). The test specimens with a thickness of 0.1–0.2 mm and a mass of 0.5–1.0 g were wrapped in copper mesh with a pore size of 125 μm. First, the copper mesh was weighed and recorded as mass one (*m*_1_). Then the test specimen was loaded into the copper mesh, weighed, and recorded as mass two (*m*_2_). Next, the wrapped specimen was put into 1000 mL of xylene solution (C_8_H_10_, liquid, purity ≥ 99.7%, Sinopharm Chemical Reagent Co., Ltd., Shanghai, China) containing 1% antioxidant 2246 (C_23_H_32_O_2_, powder, purity ≥ 99.99%, Shanghai Aladdin Biochemical Science and Technology Co., Ltd., Shanghai, China) and boiled for 8 h. Then the boiled specimens were placed in a vacuum oven at 150 °C for 3 h to remove surface solvents. Finally, the specimens were cooled and weighed as mass three (*m*_3_) [38]. The cross-linking degree (*G*) was calculated as Equation (1):

(1) $$G=\frac{m_{3}-m_{1}}{m_{2}-m_{1}}$$

where each specimen was tested three times and averaged.

#### 2.3.2. Mechanical Properties Test

All tensile strength, elongation, and flexural strength tests were carried out using a universal testing machine (KXWW, Chengde Taiding Testing Machine Manufacturing Co., Ltd., Chengde, China). The dumbbell-shaped specimens were used for tensile testing, with specific dimensions of 150 mm (total length) × 20 mm (total width) × 4 mm (thickness) × 50 mm (gage length) × 10 mm (width at the narrowest part). The rectangular specimens were used for bending strength testing, with dimensions of 80 mm × 10 mm × 4 mm. Tensile strength was evaluated using GB/T1040.2-2006 [39] at a rate of 50 mm/min. Flexural strength was measured using GB/T9341-2008 [40] with a rate of 20 mm/min.

All impact strength tests were conducted using a simple beam impact tester (KXJJ-50A, Chengde Taiding Testing Machine Manufacturing Co., Ltd., Chengde, China). The specimens for impact strength testing had the same dimensions as those for bending strength testing. The impact strength was measured using an A-notch in accordance with GB/T1843-2008 [41]. The impact strength (*a_iN_*) was determined using Equation (2):

(2) $$a_{iN}=\frac{E_{e}}{h\times b_{N}}\times 10^{3}$$

where *E_e_* is impact test fracture absorption energy; *b_N_* is the specimen’s remaining width. All specimens were prepared in five sets of replicates for the corresponding mechanical tests and averaged.

#### 2.3.3. Measurement of HDT and VST

The HDT and VST of all specimens were measured by an HDT tester (KXRW-300CL-3, Chengde Chengding Experimental Machine Manufacturing Co., Ltd., Chengde, China). The specimens for HDT testing had dimensions of 80 mm × 10 mm × 4 mm, while those for VST testing had dimensions of 20 mm × 10 mm × 4 mm. In the HDT test, the specimens were immersed in a silicone oil bath, heated at a rate of 120 °C/h, and applied with a load of 0.45 MPa. Three specimens were tested in each group, and the average value was taken. In the VST test, the specimens were immersed in a silicone oil bath and heated at a rate of 50 °C/h. At the same time, a weight of 1 kg was applied to a pressure pin. Each group had three specimens examined, and the average was taken.

#### 2.3.4. Differential Scanning Calorimetry (DSC) Test

The DSC tests were performed by means of a differential scanning calorimeter (DSC 214, Maschinenbau Group, Bavaria, Germany). Under nitrogen protection, the specimens were heated to 180 °C at rate of 10 °C/min, held for 10 min, and then the temperature was reduced to 20 °C at a rate of 20 °C/min. Finally, the heating and cooling processes were repeated for calculation of the degree of crystallinity. The crystallinity was calculated by Equation (3):

(3) $$X_{c}=\frac{{∆H}_{m}}{{∆H}_{m,100\%}\times w}\times 100\%$$

where *X_c_* (%) is the crystallinity of the HDPE, *w* is the mass content of HDPE in the composites, Δ*H_m_* (J/g) is the enthalpy of melting obtained from the DSC melting curve, and Δ*H_m_*_,100%_ (J/g) is the theoretical enthalpy of melting of the HDPE with a crystallinity of 100% in this study. The average thickness (*L_DSC_*) of wafers was calculated by Equation (4):

(4) $$L_{DSC}=\frac{2\delta }{{∆H}_{m,100\%}}\times \frac{T_{m}^{0}}{T_{m}^{0}-T_{m}}$$

where $T_{m}^{0}$ (K) is the theoretical melting temperature of the HDPE, *T_m_* (K) is the actual melting temperature of the HDPE in this study, and *δ* (J/m^2^) is the free energy of the HDPE wafer surface. The above constant parameters of HDPE are Δ*H_m_*_,100%_ = 293 J/g, $T_{m}^{0}$ = 418.6 K, and *δ* = 0.07 J/m^2^, respectively [42,43,44].

#### 2.3.5. Scanning Electron Microscopy (SEM)

The specimen was quenched by immersion in liquid nitrogen. A 1–2 mm thick slice was cut from the fracture surface. Gold was then sputter-plated on the thick slice. Finally, the micro-morphological characteristics of the specimen was observed using a field emission scanning electron microscope (S-4800, Hitachi, Tokyo, Japan) at an accelerating voltage of 5 kV.

## 3. Results and Discussion

Currently, most studies on the cross-linking of HDPE using DTBP and TAIC employ a one-step method. A few studies utilize a two-step method. However, they focus on the performance analysis of different types of polyethylene after cross-linking, without an in-depth discussion specifically on HDPE. This study adopts a two-step cross-linking approach and conducts a comprehensive analysis of the cross-linked HDPE, including its cross-linking degree, mechanical properties, thermal stability, crystallization and melting behavior, and microstructure.

### 3.1. Cross-Linking Degree

**Figure 2:**
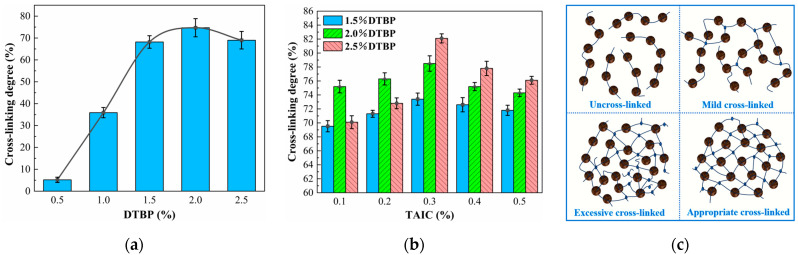
Cross-linking degree of HDPE: Different contents of DTBP and DTBP/TAIC. (**a**) Cross-linking degree (only DTBP); (**b**) Cross-linking degree (DTBP and TAIC); (**c**) Changes in molecular chains due to changes in the cross-linking degree.

The higher the cross-linking degree, the more pronounced the effect of the cross-linker on the polymer [28]. As shown in Figure 2a, the degree of cross-linking increased significantly from 5.1% to 68.2% when the DTBP concentration rose from 0.5% to 1.5%. This increase resulted from a higher number of free radicals generated by the elevated DTBP concentration, which trapped hydrogen atoms in the HDPE molecular chains [45]. At a cross-linker concentration of 2.0%, the cross-linking degree reached 74.7%. The increase in cross-linking degree slowed, as it restricted the mobility of the molecular chains and reduced the capture rate of free radicals. Further increasing the cross-linker to 2.5% decreased the cross-linking degree to 69%. This resulted from an excess of cross-linking agent producing additional free radicals. During the reaction, these free radicals consumed each other, leading to more uneven and unstable cross-linking.

**Figure 3:**
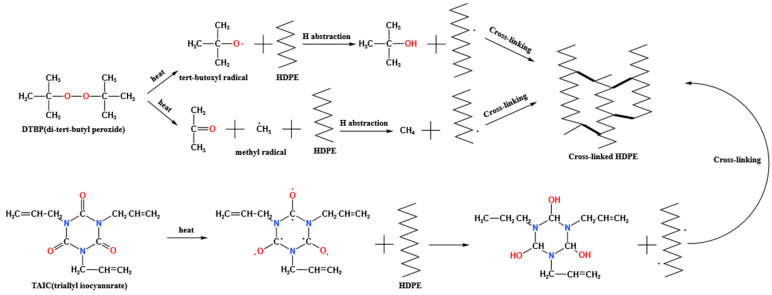
Reaction formula of DTBP- and TAIC-cross-linked HDPE.

To further increase the cross-linking degree, TAIC was introduced as a complex cross-linker. As shown in Figure 2b, the cross-linking degree initially increased and then decreased, reaching 82.1% with a DTBP concentration of 2.5% and TAIC at 0.3%. The addition of TAIC significantly enhanced the cross-linking degree compared to the pure DTBP sample. This increase resulted not only from the allyl group of TAIC directly cross-linking with HDPE but also from excess free radicals generated by DTBP binding to the double bonds on TAIC, creating new cross-linking sites [27,46]. However, the cross-linking degree began to decrease when TAIC content exceeded 0.3%. Similar to the excess DTBP scenario, an excess of cross-linking agent led to excessive free radical generation, mutual inhibition, by-product formation, and localized overcrowding of cross-linking [47]. As can be seen from Figure 2c and Figure 3, with the increase in the content and variety of cross-linking agents, the types and quantities of functional groups participating in the reaction also increased, leading to changes in the degree of cross-linking [48,49]. Therefore, control of DTBP and TAIC addition was essential for achieving optimal cross-linking effects.

### 3.2. Thermal Stability

**Figure 4:**
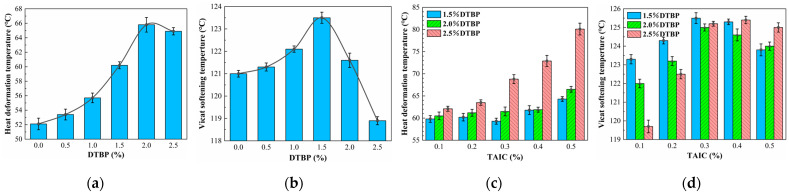
The HDT and VST of DTBP- and TAIC-cross-linked HDPE with different contents: (**a**) HDT (only DTBP); (**b**) VST (only DTBP); (**c**) HDT (DTBP and TAIC); (**d**) VST (DTBP and TAIC).

The changes in HDT and VST were different for different contents of DTBP and TAIC in cross-linked HDPE. As shown in Figure 4a, the HDT of the specimens gradually increased as the DTBP content rose from 0% to 2.0%. At 2.0% DTBP content, the HDT peaked at 65.8 °C, which was 13.7 °C higher than that of pure HDPE. This increase resulted from a greater number of cross-linking points between HDPE chains, which hindered the mobility of the molecular chains and improved the thermal deformation resistance of the specimens. However, when DTBP content reached 2.5%, the HDT decreased to 64.9 °C, primarily due to weakened cross-linking.

Regarding VST, the trend mirrored that of HDT. The maximum VST of 123.5 °C was observed at 1.5% DTBP, which was 2.5 °C higher than that of pure HDPE (121 °C). Notably, the VST began to decrease after DTBP content exceeded 1.5%, likely due to differences in testing methods and the sensitivity of VST to molecular chain mobility.

As shown in Figure 4c, the addition of TAIC further enhanced the HDT of the specimens. At a DTBP content of 2.5%, the HDT increased with rising TAIC content, reaching 80.1 °C at 0.5% TAIC, which was 15.2 °C higher than that with only 2.5% DTBP. Excessive TAIC did not reduce the HDT. This resulted from the good compatibility of TAIC with HDPE and the two-step cross-linking which facilitated a more uniform distribution of TAIC [50]. It was also possible that TAIC introduced stronger chemical bonds, such as allyl (-CH=CH-).

The VST changes of the specimens with added TAIC followed a similar trend to those with only DTBP. As shown in Figure 4d, the VST of all specimens increased to varying degrees after TAIC addition. Specifically, at 0.3% TAIC, all specimens exceeded 125 °C. When TAIC content surpassed 0.3%, the VST exhibited a slight decreasing trend. This was primarily related to the cross-linking degree and molecular chain mobility, similar to the behavior observed with only DTBP.

### 3.3. Crystallization and Melting Behaviors

**Figure 5:**
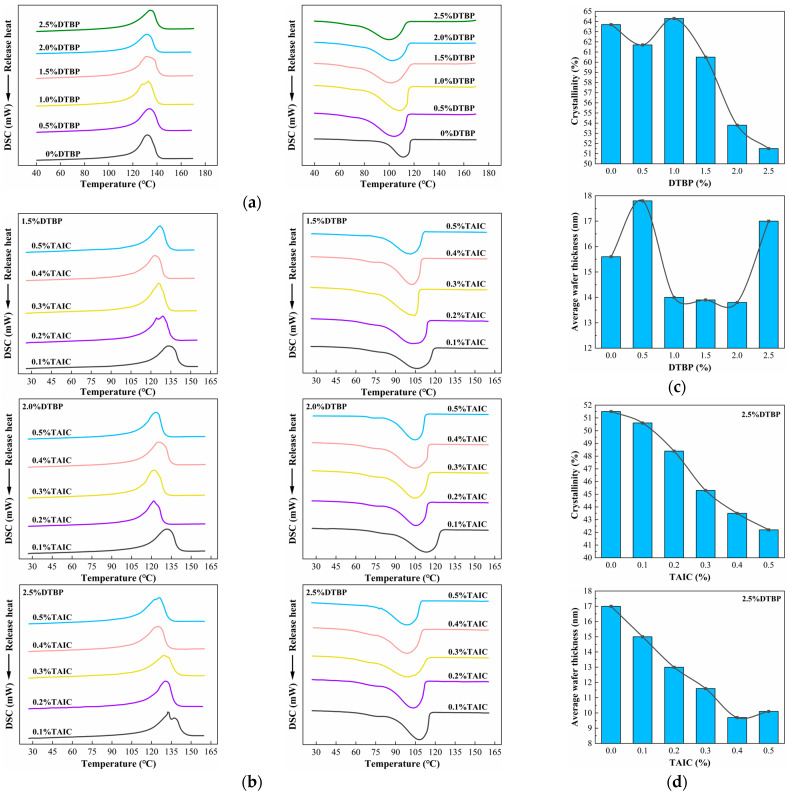
Crystallization and melting behavior of HDPE cross-linked with different contents of DTBP and TAIC: (**a**) Melting and Crystallization curve (only DTBP); (**b**) Melting and Crystallization curve (DTBP and TAIC); (**c**) Crystallinity and Average wafer thickness (only DTBP); (**d**) Crystallinity and Average wafer thickness (DTBP and TAIC).

Differences in DTBP and TAIC contents affected the crystallization and melting behavior of HDPE. As shown in Figure 5a, the melting and crystallization curve peaks of the specimens shifted leftward with increasing DTBP content. Additionally, both peaks exhibited increased width and decreased height. As shown in Figure 5b, the peak shifts were more pronounced after the introduction of TAIC, and the peaks continued to decrease. This indicated that TAIC made the cross-linking network denser, increasing crystallization difficulty.

From Figure 5c,d, it was evident that *X_c_* and *L_DSC_* decreased as cross-linker content increased. Compared to the *X_c_* of pure HDPE, the addition of DTBP reduced *X_c_* from 63.7% to 51.5%, a decrease of 12.2%. The introduction of TAIC further decreased *X_c_* to 42.2%, representing a 21.5% reduction. For *L_DSC_*, the addition of DTBP reduced it from 15.6 nm to 13.8 nm, with a less noticeable decreasing trend due to poor cross-linking uniformity. However, the trend in *L_DSC_* was particularly pronounced for specimens with TAIC, leading to a further decrease to 9.7 nm. This is because TAIC makes the distribution of the cross-linked network become more uniform and dense.

The specific parameters of melting and crystallization in Table 2 indicated that the composite system of DTBP and TAIC significantly affected the crystallization and melting behavior of HDPE. With excess DTBP, the *T_m_* and *T_c_* values fluctuated rather than continuously decreasing. In contrast, with excess DTBP, the introduction of TAIC maintained a significant downward trend in *T_m_* and *T_c_*. This suggested that TAIC promoted a uniform distribution of cross-linking reactions and facilitated the occurrence of more cross-linking reactions. A larger number of uniformly distributed cross-linking reactions resulted in decreased *X_c_* and more imperfect grains, leading to lower *T_m_* and *T_c_* [51,52].

### 3.4. Mechanical Properties

**Figure 6:**
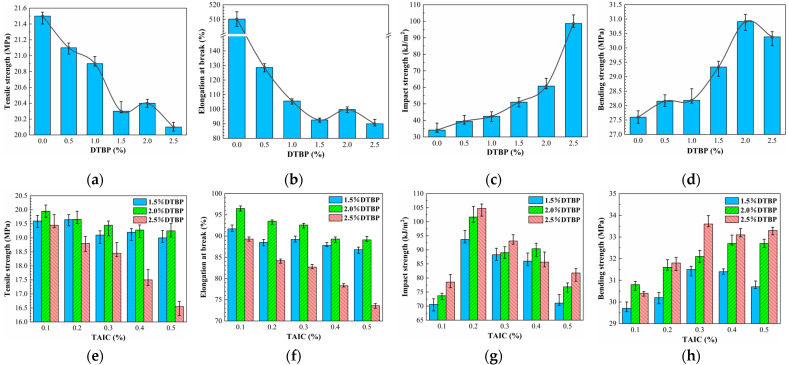
Tensile strength, elongation, impact strength, and bending strength of HDPE cross-linked with DTBP and TAIC: (**a**) Tensile strength (only DTBP); (**b**) Elongation at break (only DTBP); (**c**) Impact strength (only DTBP); (**d**) Bending strength (only DTBP); (**e**) Tensile strength (DTBP and TAIC); (**f**) Elongation at break (DTBP and TAIC); (**g**) Impact strength (DTBP and TAIC); (**h**) Bending strength (DTBP and TAIC).

As shown in Figure 6a,b,e,f, the tensile strength and elongation at break gradually decreased with increasing DTBP content. At 2.5% DTBP, the tensile strength and elongation at break reached 20.1 MPa and 90%, about 1.4 MPa and 420.2% lower than that of pure HDPE. The introduction of TAIC further decreased the tensile strength and elongation. At 2.5% DTBP and 0.5% TAIC, the tensile strength achieved minimum values of 16.55 MPa and 73.6%, representing a decrease of 3.55 MPa and 16.4% compared to 2.5% DTBP alone. This implied that TAIC restricted the free movement of molecular chains, weakening their ductility and impairing stress dispersion during tensile stretching. Additionally, with the increase in cross-linker content and the decrease in *X_c_* and *L_DSC_*, the type and quantity of cross-linking by-products rose, leading to lattice defects and decreased molecular chain ductility.

In terms of impact strength, the introduction of the cross-linking agent substantially increased the strength of HDPE. As shown in Figure 6b, at 2.5% DTBP, the impact strength reached 98.60 kJ/m^2^, which was 64.54 kJ/m^2^ higher than that of pure HDPE, reflecting a growth rate of 289.5%. This implied that increasing DTBP content raised the number of cross-linking points, enhancing specimen toughness. As shown in Figure 6e, the introduction of TAIC further improved the impact strength to 104.7 kJ/m^2^, nearly 70 kJ/m^2^ higher than that of uncross-linked specimens. This indicated that an appropriate amount of TAIC synergistically enhanced the cross-linking with DTBP, forming a more homogeneous and densely cross-linked structure, thereby improving toughness and energy absorption capacity [53]. However, at high TAIC content, the impact strength showed a decreasing trend, implying that excess TAIC weakened the synergistic effect with DTBP and may have destabilized the cross-linking mesh structure.

The variation in bending strength closely followed the cross-linking degree. As shown in Figure 6c, the highest bending strength of 30.91 MPa was achieved at 2.0% DTBP, which was 3.31 MPa higher than that of pure HDPE. The maximum bending strength of 33.6 MPa occurred at 2.5% DTBP and 0.3% TAIC, as shown in Figure 6f, coinciding with the maximum cross-linking degree. This implied that bending strength variation was related to the cross-linking degree. The decrease in *X_c_* and *L_DSC_* indicated a less uniform and more imperfect crystalline structure, which weakened the overall mechanical integrity and reduced the material’s ability to resist bending forces. Finally, bending strength decreased when excess cross-linking agent was present, due to uneven distribution of lattice defects and localized stress concentration [54].

### 3.5. SEM Analysis

**Figure 7:**
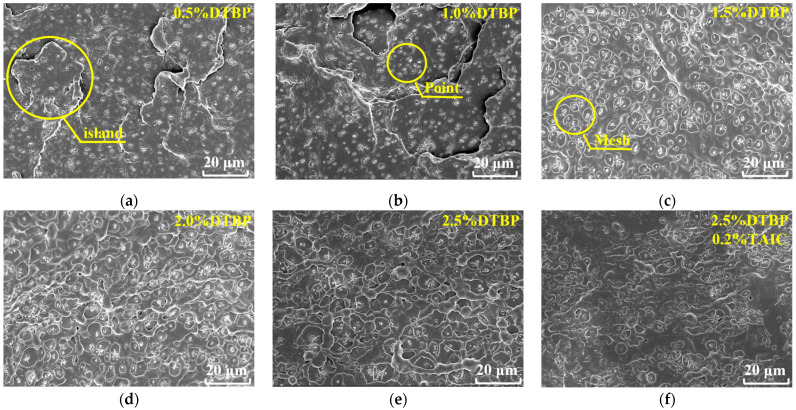

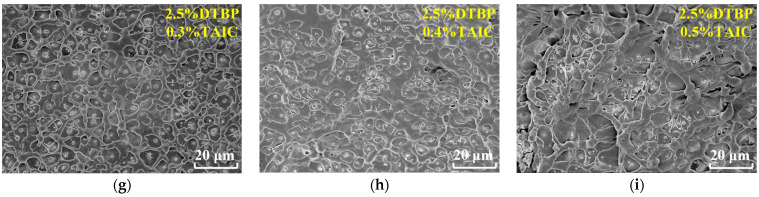
SEM images of cross-linked HDPE specimens with different DTBP and TAIC contents: (**a**) 0.5% DTBP; (**b**) 1.0% DTBP; (**c**) 1.5% DTBP; (**d**) 2.0% DTBP; (**e**) 2.5% DTBP; (**f**) 2.5% DTBP and 0.2% TAIC; (**g**) 2.5% DTBP and 0.3% TAIC; (**h**) 2.5% DTBP and 0.4% TAIC; (**i**) 2.5% DTBP and 0.5% TAIC.

As shown in Figure 7a, when the DTBP content was low, the specimen’s surface exhibited a layered island structure with a small number of dots on the islands. This indicated that a limited cross-linking reaction had occurred, with the islands representing the cross-linking regions and the white dots potentially being by-products of the reaction [27,48,49]. As shown in Figure 7b, as DTBP content increased from 0.5% to 1.0%, the sea islands became smaller and more numerous, with more pronounced stratification and an increase in white dots. This implied that the cross-linking reaction was enhanced with the rise in cross-linking agent.

In Figure 7c,d, the boundaries of the islands become less distinct, and a mesh structure appears, with white dots located in the mesh openings. The emergence of this mesh structure suggested that the cross-linking reaction reached a critical point, forming a more complex three-dimensional network. As shown in Figure 7e, the uniformity of the mesh distribution deteriorated at a cross-linker content of 2.5%. This indicated that excessive amounts of DTBP could lead to inhomogeneous reactions, which disrupted the regular arrangement of polymer chains, reduced crystallinity, weakened mechanical properties, and lowered thermal stability of HDPE.

The most significant performance change occurred when TAIC was introduced at a DTBP content of 2.5%. So, SEM tests were conducted at a DTBP content of 2.5% with varying levels of TAIC added. As shown in Figure 7e,f, with increasing TAIC content, the mesh structure distribution became more uniform, and the number of meshes improved further. Notably, at 0.3% TAIC, the specimen’s surface exhibited uniformly dispersed, regularly sized, and well-defined meshes. This implied that TAIC acted synergistically with DTBP to enhance the cross-linking reaction.

As shown in Figure 7h,i, as TAIC content continued to increase, particularly at 0.5%, the meshes on the specimen surface varied in size, became unevenly distributed, and overlapped. This resulted from excessive TAIC causing cross-linkers to overlap at certain locations. Consequently, the generated free radicals did not have sufficient time to disperse uniformly, leading to repeated cross-linking reactions in some areas. Simultaneously, cross-linking side reactions may have occurred within the sample, causing by-products to accumulate unevenly.

## 4. Conclusions

DTBP has a considerable effect on HDPE’s cross-linking properties. With an increase in DTBP level within a specific range, the cross-linking degree of HDPE steadily increases while the *X_c_* drops. At the same time, as the cross-linking degree grows, the impact strength, bending strength, HDT, and VST also improve, enhancing the mechanical characteristics and heat resistance of the cross-linked HDPE. However, when the DTBP level is too high, the cross-linking degree decreases, compromising the mechanical characteristics and heat resistance. This phenomenon is attributed to the fact that DTBP can cross-link with the molecular chain of HDPE, improving its heat resistance and mechanical characteristics. However, using too much DTBP increases cross-linking by-products while decreasing cross-linking density and homogeneity, ultimately lowering HDPE’s mechanical characteristics and heat resistance after cross-linking. As a result, in actual applications, the DTBP concentration must be properly controlled to balance the relationship between cross-linking degree and material properties.

TAIC acts synergistically with DTBP as an auxiliary cross-linker. The combination of DTBP and TAIC further enhances the cross-linking reaction of HDPE compared to DTBP alone. The most significant improvements in cross-linking degree, mechanical properties, and heat resistance are observed with 2.5% DTBP and 0.3% TAIC. Specifically, the introduction of TAIC increases the cross-linking degree to 82.1% and raises the HDT to 80.1 °C, demonstrating superior heat resistance compared to DTBP alone. Additionally, the presence of TAIC enhances the impact strength of HDPE to 104.73 kJ/m^2^, indicating a marked improvement in toughness.

These enhancements result from the good compatibility between TAIC and HDPE, which facilitates a more homogeneous and dense cross-linked network. This network improves the overall mechanical properties of HDPE. Furthermore, TAIC contributes to the stability of the cross-linked network by introducing stronger chemical bonds, further enhancing the thermal resistance and strength of the material.

## Figures and Tables

**Table 1:** Partition ratio of peroxide-cross-linked HDPE specimen.

	Component	Proportion (wt%)				
Group		A1076	DLTDP	CaSt	DTBP	TAIC
Neat HDPE		-	-	-	-	-
DTBP		0.2	0.3	0.5	0.5/1.0/1.5/ 2.0/2.5	-
DTBP and TAIC					1.5/2.0/2.5	0.1/0.2/0.3/ 0.4/0.5

**Table 2:** Measurement of melting and crystallization parameters of cross-linked specimens by DSC.

DTBP Content /(wt%)	TAIC Content /(wt%)	Enthalpy of Fusion Δ*H_m_*/(J/g)	Melting Point *T_m_*/(°C)	Enthalpy of Crystallization Δ*H_c_*/(J/g)	Crystallization Peak Temperature *T_c_*/(°C)
-	-	186.5	132.6	188.9	111.4
0.5		180.8	134.2	192.9	109.4
1.0		188.3	131.2	183.2	104.0
1.5		177.2	131.1	172.5	101.1
2.0		157.7	131.0	152.7	103.0
2.5		150.8	133.7	151.8	99.6
	0.1	148.4	132.2	152.3	100.4
	0.2	141.8	130.1	150.2	98.2
	0.3	132.6	128.2	145.3	94.3
	0.4	127.5	124.9	143.6	93.0
	0.5	123.7	125.6	140.9	93.2

## Data Availability

The data presented in this study are available on request from the corresponding author. The data are not publicly available due to privacy or ethical restrictions.

## Abbreviations

The following abbreviations are used in this manuscript:

HDPE	High-density polyethylene
PA	Polyamide
POM	Polyoxymethylene
DTBP	Di-tert-butyl peroxide
TAIC	Triallyl isocyanurate
BIPB	Bis (tertbutyldioxyisopropyl) benzenehexane
BCUP	Di-tert butyl cumyl peroxide
DLTDP	Didodecyl thiodipropionate
A1076	Antioxidant 1076
CaSt	Calcium stearate
PEXa	Peroxide cross-linking
PEXb	Silane cross-linking
PEXc	Radiation cross-linking
*X_c_*	Crystallinity
*T_m_*	Melting temperature
*T_c_*	Crystallization temperature
$T_{m}^{0}$	Theoretical melting temperature of the HDPE
G	Cross-linking degree
*a_iN_*	Impact strength
*E_e_*	Impact test fracture absorption energy
*b_N_*	Specimen’s remaining width
Δ*H_m_*	Enthalpy of melting obtained from the DSC melting curve
Δ*H_m_*_,100%_	Theoretical enthalpy of melting of the HDPE with a crystallinity of 100%
Δ*H_c_*	Enthalpy of crystallization
*L_DSC_*	Average thickness of wafers
*δ*	The free energy of HDPE wafer surface
DSC	Differential scanning calorimeter
HDT	Heat deformation temperature
VST	Vicat softening temperature
SEM	Scanning electron microscopy
